# Heyde Syndrome: A Case Report and Literature Review

**DOI:** 10.7759/cureus.7896

**Published:** 2020-04-30

**Authors:** Chidinma A Chukwudum, Saul Vera, Munish Sharma, Joseph Varon, Salim Surani

**Affiliations:** 1 Miscellaneous, Dorrington Medical Associates, Houston, USA; 2 Medicine, Dorrington Medical Associates, Houston, USA; 3 Internal Medicine, Corpus Christi Medical Center, Corpus Christi, USA; 4 Critical Care, University of Texas Health Science Center and United General Hospital, Houston, USA; 5 Internal Medicine: Critical Care, University of Texas Health Science Center and St Luke's Episcopal Hospital, Houston, USA; 6 Internal Medicine, University of North Texas, Dallas, USA

**Keywords:** heyde syndrome, aortic stenosis, gastrointestinal bleeding

## Abstract

Heyde syndrome is characterized by an association between gastrointestinal (GI) bleeding and calcific aortic stenosis (AS). Although the course of disease progression that links AS and GI bleeding has not been determined, overlaps among AS, intestinal dysplasia, and acquired von Willebrand's syndrome are thought to result in GI bleeding. Aortic valve repair in some patients has been reported to result in marked improvement or the complete resolution of signs and symptoms of Heyde syndrome. The prevalence of Heyde syndrome is higher among elderly persons than among other age groups, suggesting that a degenerative process may be a significant factor in the disease progression. This report describes a patient with Heyde syndrome, as well as a review of the current literature.

## Introduction

Heyde syndrome is characterized by a triad of aortic stenosis (AS), gastrointestinal (GI) bleed from angiodysplastic vessels, and anemia. This entity has been recognized for many years and it is has higher prevalence in the patient population but may remain unidentified. There is increasing evidence that this syndrome results in acquired von Willebrand's disease and results in loss of hemostasis. It is a rare but potentially fatal condition if not managed appropriately [1].Thus, it is prudent that physicians are more aware about Heyde syndrome so that it can be identified in a timely manner and managed appropriately. 

## Case presentation

**Figure 1:**
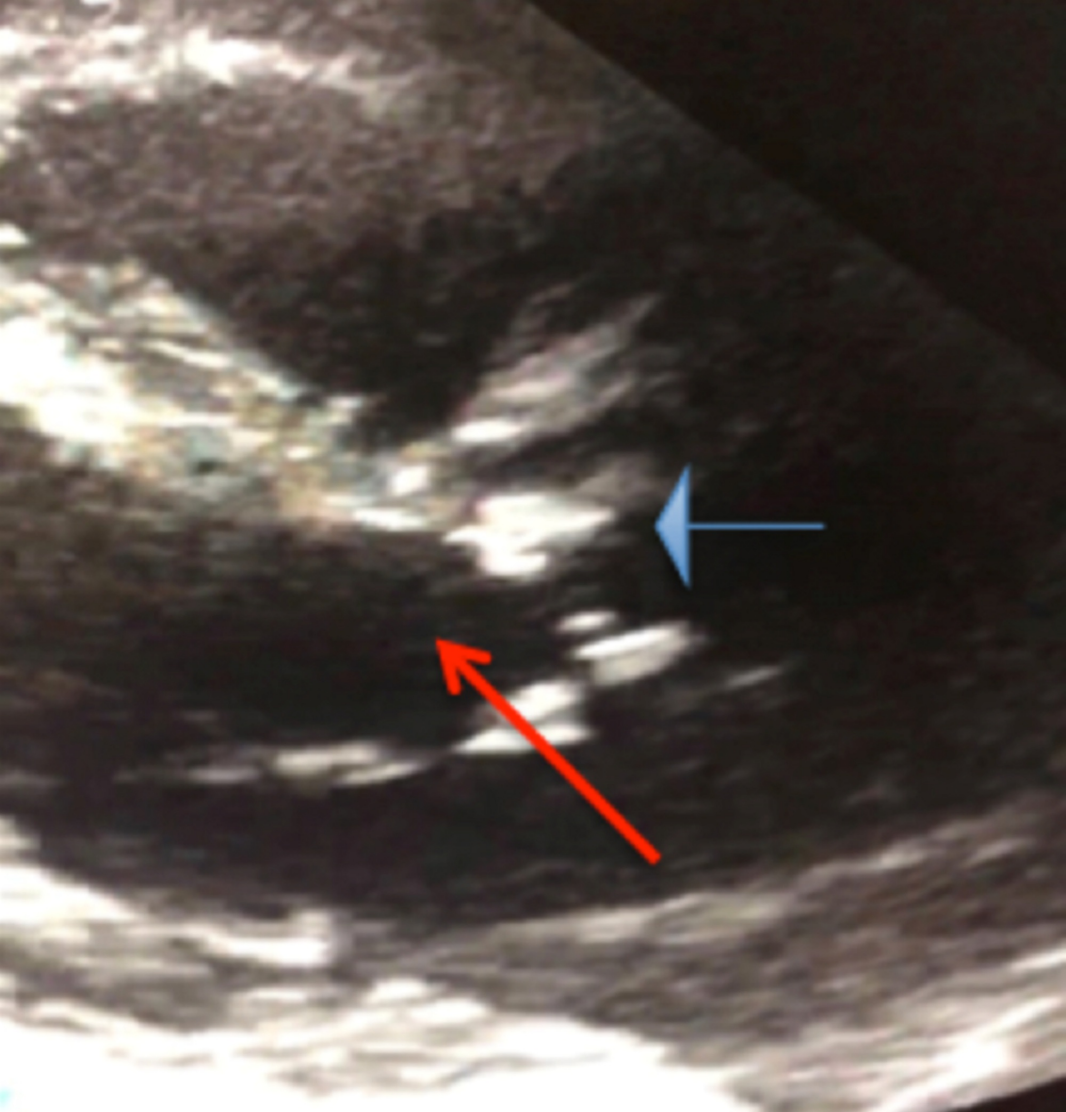
Parasternal long axis view of a two-dimensional echocardiogram, showing a calcified and stenotic aortic valve (blue arrow) and left ventricular outflow tract (red arrow).

A 61-year-old man presented to the ED after having black stools for 24 hours. This episode was preceded by the spontaneous onset of abdominal cramps, followed by dyspnea, fatigue, and light-headedness. His prior medical history included multiple hospital admissions for upper GI bleeding. There was no history of smoking, alcohol intake, or illicit drug use. On initial examination, he appeared lethargic, with a blood pressure of 101/55 mmHg, a pulse of 83 beats/minute, a respiratory rate of 16 breaths/minute, and oxygen saturation of 100% while breathing room air. He had conjunctival pallor. Cardiovascular examination revealed a systolic ejection murmur with late systolic peaking and soft second heart sounds. Laboratory tests showed a blood glucose concentration of 101 mg/dL, a white blood cell count of 4.6 x 109/L, a hemoglobin concentration of 8 g/dL, and a platelet count of 207 x 109/L. Other laboratory tests showed sodium 138 mEq/L, potassium 3.9 mmol/L, chloride 105 mmol/L, carbon dioxide 22 mEq/L, blood urea nitrogen 21 mg/dL, and creatinine 0.9 mg/dL. He was deficient in high molecular weight multimers of von Willebrand factor (vWF). Abdominal CT and upper GI endoscopy yielded negative results. A previous capsule examination of the small bowel had been negative. Two-dimensional transthoracic echocardiogram revealed a severely sclerotic aortic valve with area of 0.65 cm2 and left ventricular ejection fraction 65% (Figure 1).

The patient was treated with intravenous administration of the proton pump inhibitor octreotide as well as two units of packed red blood cells. Because of his history of chronic GI bleeding, anemia, and AS, he was diagnosed with Heyde syndrome, a diagnosis supported by the deficiency in high molecular weight multimers of vWF. 

The patient underwent a minimally invasive aortic valve replacement, with the insertion of a 27-mm tissue valve. At the present writing, three years after the procedure, the patient has been asymptomatic with no further episodes of bleeding.

## Discussion

Literature review

Although the co-occurrence of GI bleeding and AS in elderly patients was initially reported in 1958 [1-2], no consensus has yet been reached on the correlation between GI bleeding and AS in patients with Heyde syndrome [3]. GI bleeding can be precipitated by several conditions, with one of the most frequent in patients with AS being intestinal angiodysplasia (IA) [3-4]. Several studies have reported a significant association between IA and AS, whereas other studies found no association between these two conditions. For example, a large-scale epidemiological study investigating the five-year incidence of AS and GI bleeding in patients with IA in an Irish population reported that AS was significantly associated with GI bleeding [5]. In contrast, a meta-analysis found that there was no association [5]. 

Arguably, both IA and AS are due to chronic degenerative processes, the prevalence of which has been shown to increase with patient age, especially during the sixth decade of life [1-4, 6]. The simultaneous occurrence of two degenerative processes in a subset of elderly individuals and even in younger subjects is not unusual. For example, associations have been observed among chronic renal failure, risk factors for coronary arterial disease, congenital anomalies such as bicuspid valve defects, and hypertrophic cardiomyopathy (HCM) [3].

Angiodysplastic vessels that are not actively bleeding may be missed during the endoscopic examination of patients with suspected Heyde syndrome being investigated for GI bleeding. This makes their diagnosis sometimes challenging. The most common site of IA is the colon, with the cecum and ascending colon being lesion hot spots, which may be due to intermittent high wall tension over time [4]. According to Laplace's law [*T* = *p* 9 *r*/(2 9 *t*), where *T* = wall tension, *p* = pressure, *r* = radius, and *t* = wall thickness], the cecum and ascending colon have larger diameters with thinner walls, which compress the submucosal venules [4]. This is followed by congestion of the capillaries and pre-capillary sphincter, creating a hypoxic milieu and triggering neovascularization [4]. Angiogenic factors, such as vascular endothelial growth factor, have been found to play an active role in this process and may be targets for therapeutic intervention [4]. Conceptually, any process that generates a hypoxic environment, such as intrinsic or extrinsic impedance of blood flow through the vessel walls, would promote angiodysplasia, as suggested in patients with AS, low cardiac output, and HCM [3-4]. 

Low levels of high molecular weight multimers of vWF have been reported in patients without hematological disorders, but with AS and GI bleeding [3-4, 6], a condition called acquired von Willebrand disease or von Willebrand syndrome type 2A. Large multimeric vWF passing through a stenotic valve undergoes mechanical shearing, fragmenting these molecules. vWF is essential in maintaining the integrity of the blood vessels, especially when the vessel walls are compromised. Therefore, further development of AS in individuals at risk of IA, including the elderly, increases their likelihood of GI bleeding [3-4, 7]. Although rare, Heyde syndrome appears to be a point of intersection of a constellation of several otherwise independent different disease entities [3, 8-9]. 

Despite questions regarding the association between IA and AS in Heyde syndrome, several case reports have shown that surgical management by aortic valvular replacement (AVR) resulted in significant patient improvements [3-4]. AVR showed marked effects, especially in symptomatic patients, as evidenced by frequent GI bleeding and symptomatic anemia, causing repeated hospitalizations. Similar findings were observed in our patient, who underwent minimally invasive AVR and has been asymptomatic since the procedure [3, 10]. Other forms of management, such as endoscopic cauterization of ectatic vessels and pharmacological therapies, have higher recurrence rates but may be required for patients at high risk for surgery [3-4, 8-9].

## Conclusions

Heyde syndrome is an infrequently encountered clinical entity. Despite the poor understanding between the IA and AS, proper repair of aortic valve can result in significant improvement of GI bleeding and its recurrence. Thus, it is prudent that physicians be aware of this condition so that cases of Heyde syndrome are diagnosed and treated in a timely manner. 
